# Antiviral Activity of Metabolites from Peruvian Plants against SARS-CoV-2: An In Silico Approach

**DOI:** 10.3390/molecules26133882

**Published:** 2021-06-25

**Authors:** Luis Daniel Goyzueta-Mamani, Haruna Luz Barazorda-Ccahuana, Karel Mena-Ulecia, Miguel Angel Chávez-Fumagalli

**Affiliations:** 1 Vicerrectorado de Investigación, Universidad Católica de Santa María, Urb. San José s/n—Umacollo, Arequipa 04000, Peru; mchavezf@ucsm.edu.pe; 2 Departamento de Ciencias Biológicas y Químicas, Facultad de Recursos Naturales, Universidad Católica de Temuco, Ave. Rudecindo Ortega 02950, Temuco 4780000, Chile; kmena@uct.cl; 3 Núcleo de Investigación en Bioproductos y Materiales Avanzados (BIOMA), Facultad de Recursos Naturales, Universidad Católica de Temuco, Ave. Rudecindo Ortega 02950, Temuco 4780000, Chile

**Keywords:** in silico, natural products, Rutin, Peru, SARS-CoV-2, Bioinformatics, drug discovery

## Abstract

(1) Background: The COVID-19 pandemic lacks treatments; for this reason, the search for potential compounds against therapeutic targets is still necessary. Bioinformatics tools have allowed the rapid in silico screening of possible new metabolite candidates from natural resources or repurposing known ones. Thus, in this work, we aimed to select phytochemical candidates from Peruvian plants with antiviral potential against three therapeutical targets of SARS-CoV-2. (2) Methods: We applied in silico technics, such as virtual screening, molecular docking, molecular dynamics simulation, and MM/GBSA estimation. (3) Results: Rutin, a compound present in Peruvian native plants, showed affinity against three targets of SARS-CoV-2. The molecular dynamics simulation demonstrated the high stability of receptor–ligand systems during the time of the simulation. Our results showed that the Mpro-Rutin system exhibited higher binding free energy than PLpro-Rutin and *N*-Rutin systems through MM/GBSA analysis. (4) Conclusions: Our study provides insight on natural metabolites from Peruvian plants with therapeutical potential. We found Rutin as a potential candidate with multiple pharmacological properties against SARS-CoV-2.

## 1. Introduction

In the past, different pandemics have affected the health and economy of nations, such as Cholera, Spanish flu (caused by the H1N1 virus), Severe acute respiratory syndrome (SARS, caused by SARS-CoV virus), the Middle East respiratory syndrome (MERS, caused by MERS-CoV virus) [1], and, recently, the COVID-19 pandemic, caused by the novel coronavirus SARS-CoV-2. This new pandemic emerged in China in late 2019 and spread rapidly, infecting 172 million people and causing 3.7 million deaths worldwide up to June of 2021 [2,3].

The symptoms of SARS-CoV-2 infection vary from typical flu-like symptoms to severe pneumonia. Some common symptoms are fever, cough, shortness of breath or difficulty breathing, muscular aches, and possible loss of taste or smell [4]. The detection of infected individuals and the accurate diagnosis must be performed to contain the rapid disease expansion. There are several options applied routinely, such as immunological based analysis (i.e., Enzyme-linked immunosorbent assay (ELISA), lateral flow), imaging-based (i.e., computed tomography (CT)), and molecular biology-based (i.e., Reverse transcription-polymerase chain reaction (RT-PCR), loop-mediated isothermal amplification (LAMP), clustered regularly interspaced short palindromic repeats (CRISPR), and Specific high-sensitivity enzymatic reporter unlocking (SHERLOCK)) [5]. Although RT-PCR is the most accurate test among the others, it still has some challenges and issues that difficult its validation and specificity, such as false negatives and viral loads [6].

To date, the Food and Drug Administration (FDA) approved three vaccines for their emergency use (Pfizer-BioNTech, Moderna, and Janssen), and other 14 vaccines were approved for their use in at least one country [7]. According to the Our World in Data website [8], 272 million people were fully vaccinated (3.5% of the world’s population). Unfortunately, the efficacy study of the vaccines against new variants, people from various age groups, and comorbidities is still ongoing and needs validation. In addition, the FDA had only emergency-approved antiviral drugs such as remdesivir, baricitinib, and the monoclonal antibody bamlanivimab [9]. However, due to their availability and cost, there are no accessible options for mass treatment [10]. In this sense, new drugs and accessible treatments worldwide against this novel virus remains a challenge [11].

Drug repositioning is the process of discovering, validating, and marketing from previously approved drugs for other indications, which can be used as a fast-track approach guided by established target product profiles. As the new indication is built on already available pharmacokinetics and safety data, the drug development time and cost can be shortened and manufacturing issues can be better defined [12]. The study and trial of repositioning already-approved, such as the protease inhibitors lopinavir, indinavir, and ritonavir [13,14]. In the same way, the in vitro test results of these inhibitors against SARS and MERS viruses were promising but remain unclear [15]. According to the National Institutes of Health (NIH), there are 71 active studies in phases III and IV of clinical trials about the treatment and prevention of COVID-19 [16].

The SARS-CoV-2 virus belongs to the betacoronavirus genus, which is characterized by different structural proteins, such as the matrix protein (M), spike surface glycoprotein (S), hemagglutinin-esterase dimer protein, a small envelope protein (E), and *N*-terminal RNA binding domain of nucleocapsid protein (N); these proteins are attractive as therapeutic molecular targets for drug and vaccine development [17]. Furthermore, the main protease (Mpro), a protein with no human homolog, mediates the functional maturation of polypeptides related to the replication–transcription machinery assembly [18]. The Papain-like protease (PLpro) is an interferon (IFN) antagonist that constitutes a domain of the replicase polyprotein and may be active at an early stage of the replication cycle to antagonize an upstream step of IFN induction [19]. The nucleocapsid protein (N), which encapsulates the viral genome protecting it from the host cell environment and enhances the efficiency of sub-genomic viral RNA transcription, is vital for viral replication; these are promising molecular targets that are also being extensively studied and used for drug and vaccine development [20].

Natural products have traditionally played a key role in drug discovery and were the basis of most early medicines [21]. According to Newman and Cragg (2020) [22], the number of natural product-derived drugs present in the total amount of drug launchings in the market from 1981 to 2019 represented about 42%, primarily obtained from plant sources. Therefore, bioinformatics and computational approaches became crucial for rapid in silico screening of potential metabolite databases from natural sources that can be repurposed against SARS-CoV-2 for faster, safer, and cheaper drug development reported antiviral activities [23]. These simulations can be carried out against proteins, enzymes, and receptors involved in the life cycle of the SARS-CoV-2 virus.

In this context, Peru possesses 28 of the 32 current climates in the world and 84 of the 103 life zones known on earth [24]. It is considered one of the 12 megadiverse countries, with a varied flora calculated in approximately 25,000 species [25]. Thus, ~10% of the world’s flora grows in Peru, and 30% of these plants are endemic. The Peruvian population uses approximately 5000 Peruvian plants for 49 purposes or applications (1400 species are described as medicinal) [26,27]. Examples of these natural sources are the following most exported native vegetal species: *Uncaria tomentosa*, commonly known as Cat’s claw, used due to its anti-inflammatory effect, possibly due to the presence of proanthocyanidins [28]; the sap from *Croton lechleri*, commonly known as Sangre de Grado, is used due to their anti-inflammatory, antiseptic, and hemostatic properties attributed to the presence of oligomeric proanthocyanidins, taspine, and flavonoids [29,30]; and *Plukenetia volubilis L.* known as Sacha inchi, used as a nutraceutical food due to its high-quality content in triacylglycerols, polyphenols and tocopherols [31,32]. However, most studies reported crude medicinal activities, while potentially active compounds have been isolated only from a few of the plants tested [33]. In this sense, the amount and nature of experimental evidence published on active compounds isolated from Peruvian plants are still limited [34].

Considering all the properties and understanding the importance and immense value of the in silico and computer-aided drug screening, this work aimed to select potential phytochemical candidates from Peruvian plants with antiviral activity against three therapeutical SARS-CoV-2 targets. We have used bibliographic analysis, virtual drug screening, docking prediction, molecular dynamics simulations, and ligand-binding affinities, as well as the analysis of its pharmacokinetic properties.

## 2. Results

### 2.1. Literature Search

**Figure 1:**
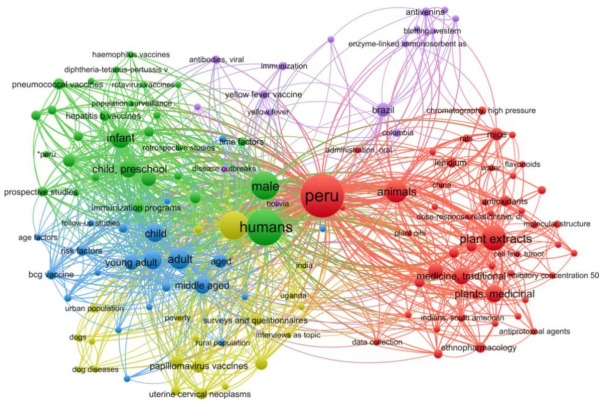
Bibliometric map created by VOSviewer based on MeSH terms co-occurrence.

In this study, a bibliographic search was performed in the PubMed database for studies that describe compounds isolated from Natural Peruvian products with antiviral properties. The search resulted in 330 articles selected, whereas the time frame distribution among the selected studies was a 70-year span (1950–2020). When conducting a co-occurrences analysis of keywords, by setting the minimum number of keyword occurrences to five, the number of keywords that meet the threshold was 134. While examining the network, it is possible to notice that the keywords were organized into five clusters; the first cluster (red color) keywords regarding the plant family “*Asteraceae*” and the genus “*Lepidium*” were found associated with terms such as “antibacterial agents”, “anti-infective agents”, and “antiprotozoal agents”. In the analysis, no “antiviral” activity related keyword was found in any of the five clusters (Figure 1). Surprisingly, only two keywords related to Peruvian plants were found in the analysis; this finding highlights a proportionally small number of studies related to anti-microbial activity compared to the vast number of described species of plants in Peru.

Antimicrobial agents with repurposing potential against viral diseases, such as teicoplanin, ivermectin, itraconazole, and nitazoxanide, have been described [35]. Considering this, compounds isolated from *Smallanthus sonchifolius* (commonly called Yacon), a plant of the family Asteraceae, native to the Andean regions, that has been studied against colon cancer, diabetes, and obesity [36], and *Lepidium meyenii* (commonly called Maca), a root native to the Andean region, that has shown neuroprotective effects, antidepressant, antioxidant, anticancer, and anti-inflammatory activities [37] were searched in the PubChem, Drug-Bank and ChEMBL databanks. In this regard, it was possible to find 14 and 26 compounds described from *S. sonchilofolius* (Table 1) and *L. meyenii* (Table 2), respectively.

### 2.2. Virtual Screening

**Figure 2:**
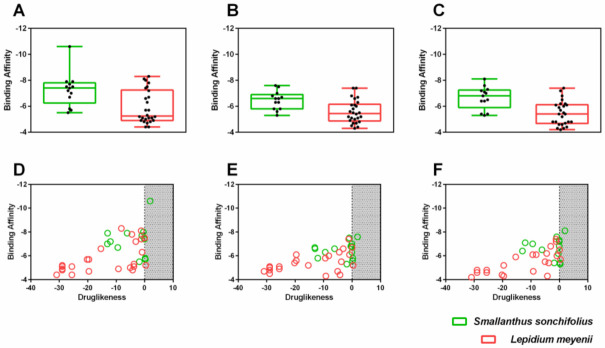
Binding affinities of the molecules selected from *S. sonchilofolius* and *L. meyenii* toward three main molecular targets of SARS-CoV2. Box plots with minimum and maximum values of binding affinities for targets. Main protease (**A**), Papain-like protease (**B**), and *N*-terminal RNA binding domain of nucleocapsid protein (**C**) are shown. Scatter plot showing binding affinities versus drug-likeness score for main protease (**D**), Papain-like protease (**E**), and *N*-terminal RNA binding domain of nucleocapsid protein (**F**) are shown.

To find potential drug candidates among the 40 compounds related to *S. sonchilofolius* and *L. meyenii*, a structure-based virtual screening approach against three major drug targets of SARS-CoV-2, namely, Papain-like protease, *N*-terminal RNA binding domain of nucleocapsid protein, and main protease, was performed. Results have shown that *S. sonchilofolius* compounds presented statistically higher binding affinity when compared to *L. meyenii* compounds in the three-drug targets (Figure 2A–C). Complementary, the calculation of the drug-likeness score using OSIRIS DataWarrior was also performed, and the results have shown that only one compound, Rutin (PubChem ID: 5280805), presented a positive score (Figure 2D–F).

### 2.3. Molecular Dynamics Simulation Analysis

The potential drug prediction was conducted to the active site by docking prediction in the PATCHDOCK server. The snapshot obtained from 50 ns of NPT simulation was used for the calculation of the thermodynamics parameter.

The first analysis was the root mean squared deviation (RMSD) of the backbone; this defined the similarity between two superimposed structures of backbone from PLpro, Mpro *N*-terminal RNA binding domain of nucleocapsid protein. In Table 3, the average RMSD values report a slight variation during the trajectory in the case of Mpro without ligand (0.18 ± 0.04 nm). Indeed, the Mpro-rutin system has an average RMSD value of 0.14 ± 0.01 nm, lower than Mpro without ligand. Herein, the ligand contributed to the structural stability of Mpro begin 25 ns until the end of the simulation. In PLpro, the average RMSD values indicated that the protein was less stable when it was bound to the ligand. Therefore, it seems to achieve conformational changes in the PLpro structure. However, the *N*-terminal RNA binding domain of nucleocapsid protein with Rutin shows more structural stability.

The radii of gyration (Rg) analysis helped us verify the protein structures’ compaction, where the average value for Mpro was similar in the two situations. As for the compaction of PLpro, this is reduced if it is binding with the rutin molecule. On the other hand, if we see the RG value of the *N*-rutin system, the compaction of this protein is increased. In summary, these average RG values of the MD simulation showed that the rutin molecule has a more significant effect on the PLpro structure than the other proteins.

**Figure 3:**
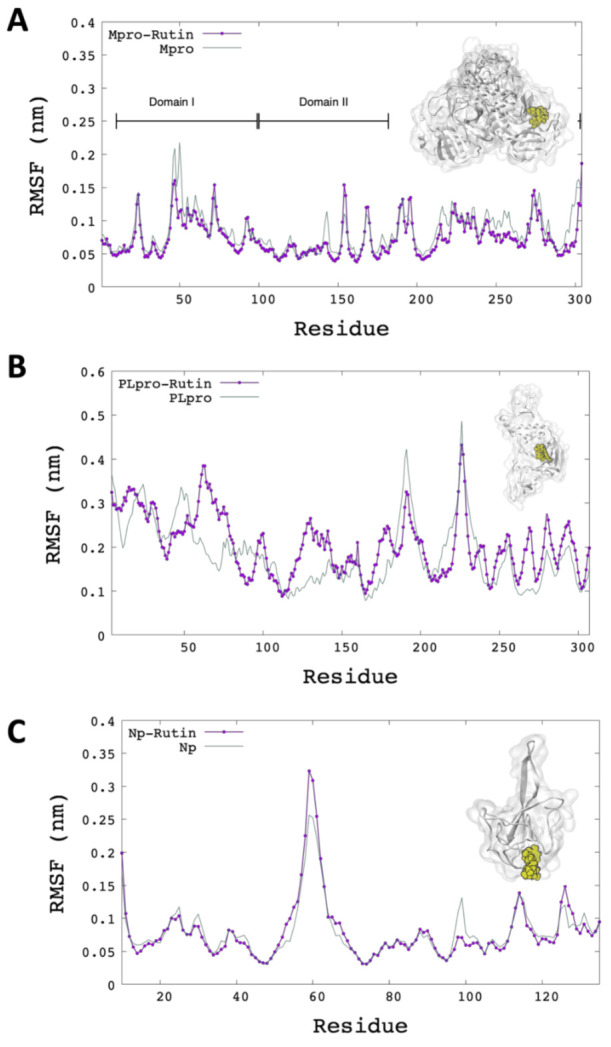
RMSF of C-alpha atoms per residue of protein with ligand (purple) and without ligand (green). (**A**) RMSF for Mpro; (**B**) RMSF for PLpro; (**C**) RMSF for N protein.

Additionally, the average root mean squared fluctuations (RMSF) per residue of the last 5 ns are represented in Figure 3. The Mpro-rutin system showed low fluctuations concerning the system without ligand, for example, domain I of Mpro showed the most significant reduction in fluctuations per residue (Mpro-rutin). This result can signal that the Rutin contributes to the stability of the protein (Figure 3A). However, in the Plpro-rutin system, high fluctuations per residue in the protein are observed; Figure 3B represents the RMSF per residue of one of the three subunits of the Plpro complex docking. Figure 3C shows the RMSF of the N protein without significant fluctuations.

### 2.4. MM/GBSA Estimation

We analyzed the MM/GBSA considering the last 500 snapshots from MD simulations to estimate the binding free energy between the complexes studied. The results in Table 4 indicate the estimation of MM/GBSA in kcal/mol. In the Mpro-rutin system, there is a more significant energy contribution by the van der Waals energies (−53,481 kcal/mol), while Plpro-rutin and *N*-terminal RNA binding domain of nucleocapsid protein-rutin systems were −21.713 kcal/mol and −34.342 kcal/mol, respectively. Furthermore, there is a more significant contribution for the Plpro-rutin system (−47.024 kcal/mol) than the other systems regarding electrostatic energy contribution. Furthermore, the contribution of gas-phase energy to the total net energy of the system was significantly high in all systems studies. Finally, the total binding energy in Mpro-rutin was better than in other systems (−40.293 kcal/mol). However, in all systems is possible the action of Rutin.

Considering the results of the molecular dynamics simulations and the energy estimation by the MM/GBSA method, we found that the Mpro-rutin system was more stable than PLpro and *N*-terminal RNA binding domain of nucleocapsid protein. Thus, we tested an HIV protease inhibitor (Lopinavir) in order to compare it with the best candidate obtained by virtual screening of this work.

The molecular structure of Lopinavir was downloaded from Protein Data Bank by the access code PDB ID: AB1. The ACPYPE server generated the topologies of Lopinavir in the AMBER force field like the methodology for Rutin.

**Figure 4:**
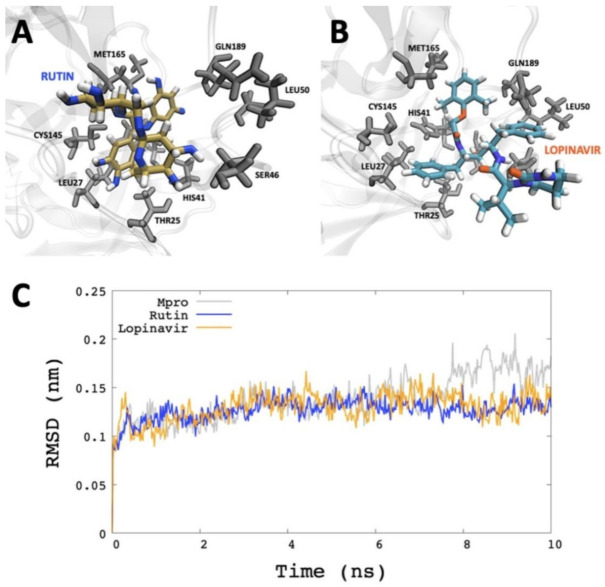
Comparison of molecular dynamics simulation trajectory between Rutin and Lopinavir. (**A**) SARS-CoV-2 Mpro with Lopinavir in the last frame of the MD simulations. (**B**) SARS-CoV-2 with Rutin in the last frame of the MD simulations. (**C**) RMSD plotting of backbone of SARS-CoV-2 Mpro.

In principle, molecular dynamics simulation was applied to analyze the behavior of the Mpro-lopinavir system. The same computational details proposed in the methodology for molecular dynamics simulation of Mpro-rutin were used. Figure 4 shows the RMSD analysis, which indicates that both ligands show similar effects on the structural stabilization of Mpro. In addition, we were able to analyze the binding free energy of Mpro-lopinavir through MM/GBSA. Our results indicated that rutin showed better binding free energy (−40.293 ± 5.740 kcal/mol) than Lopinavir (−32.7810 ± 1.8461 kcal/mol).

## 3. Discussion

Peru is one of the privileged countries with incredible biodiversity. However, there are relatively few studies on the antimicrobial and antiviral effect of metabolites from these sources. Based on the richness of Peruvian plants, the present study aimed to perform virtual screening of various isolated natural compounds against SARS-Cov-2 therapeutic targets. Our results showed the promising effect of a compound found on *S. sonchilofolius*, called Rutin, against three SARS-CoV-2 targets: Main Protease, Papain Like Protease, and *N*-terminal RNA binding domain of the nucleocapsid protein. Rutin is a flavonol widely present in various fruits and vegetables [62], with antioxidant, anti-inflammatory, and antiviral effects [63].

Previous reports have shown the potential use of plants and isolated compounds against SARS-CoV targets and other human coronaviruses. Terpenoids and lignoids isolated from *Chamaecyparis obtuse var*. *formosana Hayata*, *Juniperus formosana Hayata*, and *Cryptomeria japonica* (*Thunb. ex L.f.*) *D.Do* showed the ability to inhibit viral replication [64]; the flavonoids rhoifolin, herbacetin, and pectolinarin, from a flavonoid library, showed noteworthy inhibitory activity against SARS-CoV virus [65]; and epigallocatechin gallate and gallocatechin gallate also inhibited SARS-CoV 3CLpro. However, selecting compounds for further clinical assessment should be carefully considered, and it is crucial that in vitro and in silico experimental evidence be conclusive [66].

*Smallanthus sonchifolius*, also known as yacón, is a perennial hemicryptophyte tuberous shrub native to the South American Andes, widely cultivated for its nutraceutical and medicinal properties [67]. Several studies had shown that the consumption of yacón has improved parameters related to type II diabetes, improved insulin production and decreased blood glucose levels. Furthermore, the anti-inflammatory and antioxidant activities of organic and aqueous extracts of yacón tubers and aerial parts have been described [68]. Furthermore, note the antibacterial potential of the constituents of *S. sonchifolius* against methicillin-resistant *Staphylococcus aureus* [69], *Bacillus subtilis*, and *Pyricularia oryzae* [41].

In recent years, computational simulations allowed the improvement of structural modelling of macromolecules. This study compared the molecular dynamics of Rutin against three SARS-CoV-2 therapeutic targets (Mpro, Plpro and *N*-terminal RNA binding domain of nucleocapsid protein). Mpro is a homodimer enzyme with a catalytic dyad composed of Cys145 and His41 [70]. Our results show that Rutin has remained interacting in the catalytic dyad of Mpro with Asn142, Met49, His41, and Asp187. Our results agree with Jin and collaborators’ findings, where they found that the N3 inhibitor interacts with the residues Asn142, Met49, His41, Cys145, Gln189, Val3, Leu4, Leu27, and Thr25 [71]. In this computational approach, the Rutin showed a high probability of binding to Mpro.

To date, there is no drug specifically formulated for the treatment of Covid-19 and approved by the FDA. Therefore, specific candidates were repurposed from their actual use among the best results, such as the antiretrovirals boceprevir, asunaprevir, narlaprevir, paritaprevir, and telaprevir, used for the hepatitis C and Lopinavir for HIV treatments, whose effect on the protein Mpro are still being evaluated [72,73]. In our study, when we compared the inhibitory effect of Lopinavir to Rutin, we observed a similar positive effect between both compounds within the Mpro pocket, confirming the potential of Rutin against SARS-CoV-2 Mpro.

In parallel, it is reported that among candidates in clinical trials, such as protease inhibitor GC376 and ketone-based inhibitors showed promising action due to their interaction with dyad catalytic (His 41 and Cys145) [74,75,76].

On the other hand, Plpro is a therapeutic target composed of three protein subunits. Previous studies of the crystal structure of Plpro bound to an inhibitor showed that the binding site was composed of Tyr268, Asp164, and Gln269 [77]. The molecular dynamics simulation of the Plpro-rutin complex demonstrated that Rutin maintains an interaction with the Tyr268 and Gln269 residues comparable with experimental methods. Regarding the *N*-terminal RNA binding domain of nucleocapsid protein, we note that the Rutin maintains a coupling that allows the fluctuations reduction in this protein domain. In all cases, the estimation of the MM/GBSA showed favorable binding free energy in the complexes analyzed, where the best energy is observed for the Mpro-rutin. In addition, this system was the most stable system during the molecular dynamics simulations.

## 4. Materials and Methods

### 4.1. Literature Search Strategy and Data Collection

The bibliographic extraction was performed from the National Center for Biotechnology Information (NCBI) databases. PubMed (https://www.ncbi.nlm.nih.gov/pubmed/, accessed on 23 December 2020) is a free resource database and provides uniform indexing of biomedical literature, the Medical Subject Headings (MeSH terms), which form a controlled vocabulary or specific set of terms that describe the topic of a paper consistently and uniformly [78]. In this regard, MeSH terms were employed in the string query to improve the search accuracy. The data set was retrieved from PubMed on 23 December 2020, based on the following search string: “Peru” [MeSH Terms] AND “Natural products” [MeSH Terms]. To search among the results for Peruvian natural products with antiviral proprieties, the co-occurrence network map of MeSH terms was created using the VOSviewer server (Version 1.6.16) [79].

The simplified molecular-input line-entry system (SMILE) of compounds previously described in the natural products selected in the previous step was searched and retrieved from PubChem [80], DrugBank [81], or ChEMBL [82] servers. The following physicochemical properties, Total Molecular weight (MW), octanol/water partition coefficient (iLOGP), Number of H-Bond acceptors (HBAs), Number of H-bond Donors (HBDs), and the molecular polar surface area (TPSA), for each compound were calculated within the Osiris DataWarrior v05.02.01 software [83].

### 4.2. Virtual Screening

The FASTA sequences of the drug targets Papain-like protease (PLpro) (PDB ID: 6W9C), *N*-terminal RNA binding domain of nucleocapsid protein (N) (PDB ID: 6M3M) and main protease (Mpro) (PDB ID: 6LU7) of SARS-CoV-2 were retrieved from Protein Data Bank (https://www.rcsb.org, accessed on 23 December 2020), and subject to automated modeling in SWISS-MODEL [84]. Furthermore, the compounds were imported into OpenBabel within the Python Prescription Virtual Screening Tool (PyRx) [85] and subjected to energy minimization. PyRx performs structure-based virtual screening applying docking simulations using the AutoDock Vina tool [86], whereas the drug targets were uploaded as macromolecules. For the analysis, the search space encompassed the whole of the modeled 3D models; and the docking simulation was then run at exhaustiveness of 8 and set to only output the lowest energy pose.

The Osiris DataWarrior software was employed to calculate the drug-likeness score of each compound; the calculation is based on a library of ~5300 substructure fragments and their associated drug-likeness scores. This library was prepared by fragmenting 3300 commercial drugs as well as 15,000 commercial non-drug-like Fluka compounds. Furthermore, the potential tumorigenic, mutagenic, and irritant action of each compound was predicted by comparison to a precompiled fragment library derived from the RTECS (Registry of Toxic Effects of Chemical Substances) database [83].

### 4.3. Molecular Dynamics Simulations and Molecular Mechanics Generalized Born Surface Area Calculations

The protein structures downloaded were reviewed in a Text Editor, and the molecules that are not necessary for calculations were deleted. Likewise, based on homology modeling in the Swiss-Model server (https://swissmodel.expasy.org, accessed on 12 January 2021), it was possible to add the missing residues to complete the protein structures. In the case of PLpro, the Zn ions’ coordination with the SG atom of Cys270, Cys224, Cys189, Cys192, and Cys226 was considered.

The automatic generation topologies with the AMBER force field for the rutin molecule were obtained from ACPYPE server [87]. It was selected the charge method semi-empirical AM1-BCC, parameterized to reproduce HF/6-31G * RESP charges. Consequently, molecular dynamics (MD) simulations were carried out in GROMACS v. 2020 [88]. We considered AMBER99SB-ILDN force field, TIP4P water model, and ions to neutralize the system. The conditions of simulations consisted of three steps: the first was the minimization with the steepest descent algorithm for 50,000 steps, the second was the MD of the equilibrium in the NVT canonical ensemble with V-rescale thermostat at 309.65 K with a time of calculation of 10 ns, and the third step was the MD production without restraint condition in the NPT isobaric-isothermal ensemble with V-rescale thermostat at 309.65 K and Parrinello–Rahman barostat at 1 bar of reference pressure by 50 ns. The thermodynamic parameters were analyzed using the Gromacs tools and the plotting graph realized with Gnuplot v5 package [89].

To determine the binding free-energy of receptor–ligand, we used the Molecular Mechanics Generalized Born Surface Area (MM/GBSA) using gmx MMPBSA v1.4.1 [90] tool based on MMPBSA. py [91] From AmberTools20 suite. The molecular visualization of the interaction of the complex receptor–ligand structure was carried out using the VMD v1.9.4 (Visual Molecular Dynamics) software [92], and the 2D diagrams of receptor–ligand were visualized using the PoseView server [93].

## 5. Conclusions

This work has analyzed many data reported in PubMed data based on natural compounds from Peruvian plants with antiviral activity. After screening, our results showed that *Smallanthus sonchilofolius* and *Lepidium meyenii* were the most outstanding options due to their antibacterial, anti-infective, anti-inflammatory, antineoplastic, antioxidant, and antiprotozoal effect. Considering these native plants, we found a total of 40 reported compounds, where the Rutin compound showed high binding affinity against three therapeutic targets of SARS-Cov-2: Main Protease, Papain Like Protease, and *N*-terminal RNA binding domain of the nucleocapsid protein. To complement this work, computational simulation methods with explicit solvent were employed. Our results demonstrated that the Mpro-rutin system had the best coupling during the molecular dynamics simulation. Moreover, the MM/GBSA free energy calculations showed that the Mpro-rutin complex had the highest energy. Therefore, we propose the potential use of Peruvian native plants, like *Smallanthus sonchilofolius*, for further in vitro and in vivo studies in search of new alternatives in the treatment of COVID-19 that could be used in future drug formulations.

## Figures and Tables

**Table 1:** Natural compounds description of *S. sonchilofolius*.

PubChem ID	Structure	Name	Part of the Plant/Extract	TMW	cLogP	HBA	HBB	TPSA	References
689043	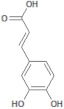	Caffeic acid	Roots/Methanolic extract	180.16	0.78	4	3	136.56	[38]
1794427	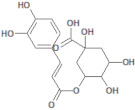	Chlorogenic acid	Roots/Methanolic extract	354.31	−0.768	9	6	245.29	[39]
445858	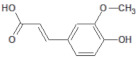	Ferulic acid	Roots	194.19	1.06	4	2	152.47	[40]
131753040	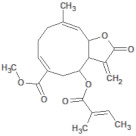	Sonchifolin	Leaves/Methanolic extract	374.43	4.06	6	0	294.45	[41]
101324862	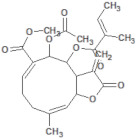	Polymatin B	Leaves/NA	432.47	3.52	8	0	333.2	[42]
92043370	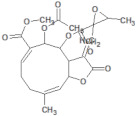	Uvedalin	Leaves/dichloromethane extract	448.47	2.09	9	0	335.55	[41]
101250074	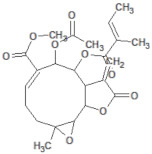	Fluctuanin	Leaves/dichloromethane extract	448.47	2.09	9	0	335.55	[43]
73062	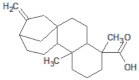	Kaurenoic acid	Leaves/NA	302.46	4.12	2	1	223.44	[44]
370	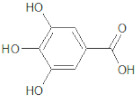	Gallic acid	Leaves/NA	170.12	0.11	5	4	116.41	[45]
5280805	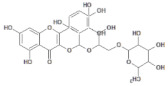	Rutin	Leaves/NA	610.52	-1.26	16	10	397.71	[45,46,47,48]
5281672	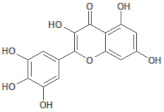	Myricetin	Leaves/NA	318.24	1.14	8	6	208.29	[49]
5280863	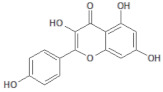	Kaempferol	Leaves/NA	286.24	1.84	6	4	195.59	[50]
5280343	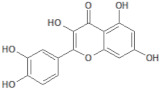	Quercetin	Leaves/NA	302.24	1.49	7	5	201.94	[51]

TMW: Total Molecular weight; iLOGP: octanol/water partition coefficient, HBAs: Number of H-Bond acceptors; HBDs: Number of H-bond Donors; TPSA: molecular polar surface area.

**Table 2:** Natural compounds description of *L. meyenii*.

PubChemID	Structure	Name	Part of the Plant/Extract	TMW	cLogP	HBA	HBB	TPSA	References
656498	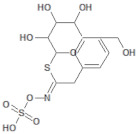	Glucotropaeolin	Hypocotyl, roots/ethanol extract	409.44	−0.75	10	5	270.57	[52,53]
6602400	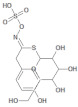	Glucosinalbin	Hypocotyl/methanol extract	425.43	−1.09	11	6	276.92	[53,54]
5485207	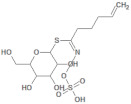	Glucobrassicanapin	Hypocotyl/methanol extract	387.43	−0.559	10	5	262.87	[53,55]
5317667	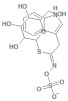	Glucobrassicin	Hypocotyl/methanol extract	447.46	−1.957	11	5	295.92	[53,55]
11198769	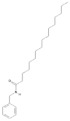	*N*-Benzylpalmitamide	Hypocotyl/hexane-ethanol extract	345.57	7.43	2	1	321.13	[56]
220495	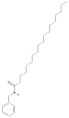	*N*-Benzyloctadecanamide	Hypocotyl/hexane-ethanol extract	373.62	8.34	2	1	348.65	[57]
68742556	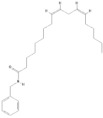	*N*-Benzyl-linoleamide	Hypocotyl/hexane-ethanol extract	369.59	7.84	2	1	346.61	[58]
68741582	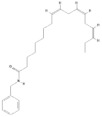	*N*-Benzyl-(9z,12z,15z)-octadecatrienamide	Hypocotyl/hexane-ethanol extract	367.58	7.59	2	1	345.59	[59]
5280450	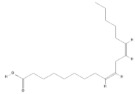	Linoleic acid	Hypocotyl/ether extracts	280.45	6.47	2	1	267.98	[48,60]
445639	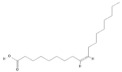	Oleic acid	Hypocotyl/ether extracts	282.47	6.72	2	1	269	[48,61]
71386083	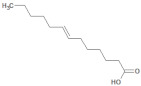	7-Tridecanoic acid	Hypocotyl/hexane extracts	212.33	4.45	2	1	200.2	[61]

TMW: Total Molecular weight; iLOGP: octanol/water partition coefficient, HBAs: Number of H-Bond acceptors; HBDs: Number of H-bond Donors; TPSA: molecular polar surface area.

**Table 3:** Average values of the parameters root mean squared deviation (RMSD), and radii of gyration (Rg) analyzed for all trajectory (50 ns).

	Mpro	Mpro *	Plpro	PLpro *	N	N *
RMSD (nm)	0.18 ± 0.04	0.14 ± 0.01	0.52 ± 0.12	0.58 ± 0.14	0.17 ± 0.04	0.13 ± 0.02
RG (nm)	2.55 ± 0.01	2.55 ± 0.01	3.28 ± 0.05	3.24 ± 0.05	1.52 ± 0.01	1.53 ± 0.01

* Protein with Rutin.

**Table 4:** Binding free energies estimated for SARS-CoV-2 targets with Rutin by MM/GBSA method.

Energy Component	Mpro-Rutin			Plpro-Rutin			*N*-Rutin		
	Average	Std Dev	Std Err of Mean	Average	Std Dev	Std Err of Mean	Average	Std Dev	Std Err of Mean
VDWAALS	−53.481	4.310	0.193	−21.713	6.213	0.278	−34.342	3.510	0.157
EEL	−33.983	10.924	0.488	−47.024	23.706	1.059	−19.027	7.432	0.332
EGB	53.900	7.243	0.324	60.491	18.393	0.822	31.047	5.453	0.244
ESURF	−6.730	0.402	0.018	−3.451	0.816	0.036	−4.684	0.371	0.017
Δ Ggas	−87.463	11.289	0.504	−68.737	22.061	0.986	−53.370	8.381	0.374
Δ Gsolv	47.170	7.075	0.316	57.041	18.012	0.805	26.363	5.246	0.234
Δ TOTAL	−40.293	5.740	0.256	−11.697	6.628	0.296	−27.007	4.115	0.184

VDWAALS = Van Der Waals energy; EEL = Electrostatic energy; EGB = Electrostatic contribution free energy calculated by Generalized Born; ESURF = nonpolar contribution to the solvation free energy; Δ Ggas = estimated binding free energy phase gas; Δ Gsolv = estimates binding free energy solvent; Δ TOTAL = Estimated binding free energy. Values of energy in kcal/mol.

## Data Availability

Not applicable.

## Sample Availability

Samples of the compounds are not available from the authors.
